# Submesoscale feedback suppresses marine heatwaves

**DOI:** 10.1126/sciadv.aei4624

**Published:** 2026-10-21

**Authors:** Jihai Dong, Wenjin Sun, Baylor Fox-Kemper, Zhiyou Jing, Changming Dong

**Affiliations:** ^1^ State Key Laboratory of Climate System Prediction and Risk Management, Nanjing University of Information Science and Technology, Nanjing, Jiangsu, China.; ^2^ School of Marine Sciences, Nanjing University of Information Science and Technology, Nanjing, Jiangsu, China.; ^3^ State Key Laboratory of Severe Weather Meteorological Science and Technology (LaSW), Chinese Academy of Meteorological Sciences, China Meteorological Administration, Beijing, China.; ^4^ Department of Earth, Environmental, and Planetary Sciences, Brown University, Providence, RI, USA.; ^5^ State Key Laboratory of Tropical Oceanography, South China Sea Institute of Oceanography, Chinese Academy of Sciences, Guangzhou, Guangdong, China.

## Abstract

Marine heatwaves (MHWs) are devastating extreme ocean temperature events whose frequency and severity have surged under global warming, threatening marine ecosystems and fisheries. Yet, the fundamental mechanisms that control their intensity and persistence remain incompletely understood. Here, using a submesoscale-permitting global model simulation validated by long-term observational and reanalysis data, this study reveals a previously overlooked submesoscale feedback that exerts a critical control on MHW severity. It is demonstrated that the submesoscale-induced vertical heat flux suppresses the intensity and persistence of atmosphere-driven MHWs in winter via a negative feedback mechanism. Enhanced air-sea net heat flux strengthens stratification and shoals the mixed layer, weakening submesoscale vertical heat transport by 19.4%, reducing MHW intensification by 18.1%, and accelerating decay by 21.7%. This missing regulatory mechanism indicates that models ignoring the submesoscale feedback likely overestimate MHW severity, with critical implications for future climate projections.

## INTRODUCTION

Compared with atmospheric temperature, upper ocean temperature tends to vary slowly due to its relatively high heat capacity and the deep mixed layer. Ocean ecosystems and life can be severely and irreversibly affected when seawater experiences extreme temperature anomalies. Thus, the concept of marine heatwaves (MHWs) was proposed to describe the extreme warm anomalies (*1*, *2*). As a type of marine disaster, MHWs have become an important disturbance factor, reshaping ocean ecosystems.

MHWs can trigger species distribution shifts, ecosystem restructuring, and economic losses in fisheries by exceeding species thermal tolerance thresholds or altering interspecies interactions (*3*–*5*). For example, the 2010–2011 MHW in Shark Bay, Western Australia, led to seagrass die-off, the deterioration of green sea turtle health, and functional extinction of kelp forests (*6*). Another event, the 2013–2015 “Blob” in the northeastern Pacific, resulted in a drastic decline in phytoplankton, harmful algal blooms, and massive mortality of high trophic-level species (*7*–*9*). Because of global warming, the frequency, intensity, and duration of MHWs have notably increased over the past century (*4*, *10*–*12*), and the return period of extreme MHW events has been continuously shortened, reaching the highest levels on record in 2023–2024 (*13*, *14*).

The generation of MHWs exhibits remarkable multiscale coupling characteristics and is co-driven by atmospheric and oceanic processes (*15*). From the perspective of air-sea interaction, regional high-pressure systems can induce anomalies in air-sea heat fluxes by reducing wind speed (hence reducing latent and sensible fluxes) and enhancing shortwave radiation (*16*, *17*). Atmospheric teleconnections, on the other hand, can modulate the evolution of heatwaves on a larger scale (*9*, *18*). For instance, the El Niño-Southern Oscillation and Indian Ocean Dipole affect heatwaves in tropical regions, while the Pacific Decadal Oscillation, North Pacific Oscillation, and North Atlantic Oscillation influence those in mid-to-high latitudes (*15*, *19*). From the perspective of oceanic dynamic processes, ocean circulation and mesoscale eddies dominate the onset and decay of MHWs in most regions through heat flux convergence and advection processes (*20*–*23*).

The movement of the ocean is multiscale, and the spatial scales of the motions span from large-scale circulation of thousands of kilometers to small-scale turbulence of less than a meter. Among these motions, submesoscale processes with spatial scales of 0.1 to 10 km have become a hot topic in the past two decades (*24*, *25*). Submesoscales are ubiquitous in the upper ocean in the form of eddies, fronts, and filaments (*26*, *27*). Different from large (∼1000 km) and mesoscale (∼100 km) processes, submesoscales are ageostrophic due to a weakening relative role of the Earth’s rotational effect at smaller scales, and relatedly can induce strong vertical motions of order of O(100) m/day (*28*–*33*). Numerous studies have shown that the submesoscale-induced vertical heat flux is predominantly upward, and its magnitude can be comparable to the sea surface heat flux, indicating a critical role in the upper ocean heat budget (*26*, *34*–*39*). It is noteworthy that submesoscale motions in the upper ocean are more active in winter, with a deeper mixed layer providing more available potential energy for submesoscale turbulence to grow (*39*–*46*).

Given their critical role in the upper-ocean heat budget and tendency toward restratification (i.e., warming the surface), submesoscale activities are hypothesized to contribute to the evolution of MHWs in winter. However, to date, their contribution remains unclear. In this work, we diagnose the heat budget of MHWs using LLC4320, a global high-resolution MITgcm simulation with a nominal horizontal resolution of 1/48° that covers September 2011 to November 2012 (details in Materials and Methods). On the basis of this analysis, we demonstrate that submesoscale-induced heat flux can suppress MHW severity via a negative feedback mechanism, potentially playing an important role in MHW evolution and prediction.

## RESULTS

### MHWs in the winter months of 2012

Given that submesoscales are most active in winter, we only focus on the winter season here (January, February, and March in the Northern Hemisphere; July, August, and September in the Southern Hemisphere). Different from many other methods for defining MHWs, the definition adopted here takes into account their horizontal spatial extent (see Materials and Methods). So, an MHW event is defined as a region encompassing all adjacent grid points identified as heatwaves.

Figure 1 presents the number and intensity of heatwaves in 2012 derived from the sea surface temperature (SST) data of the high-resolution LLC4320 simulation, compared with those obtained from the observational OISST data product (drawn from a combination of satellite, ship, and Argo observations using optimal interpolation methods; see Materials and Methods). During this period, regions with high heatwave occurrences are mainly concentrated in the North Pacific, North Atlantic, and Southern Ocean (Fig. 1). The spatial distributions indicate that the MHW event counts simulated by LLC4320 are highly consistent with the observations (Fig. 1, A and B). Moreover, a further comparison indicates that the regions with high MHW intensity are generally consistent between LLC4320 and OISST, although there are differences in the specific distribution locations (Fig. 1, C and D). Here, MHW intensity is defined as the spatially and temporally integrated SST anomaly relative to the climatology over the identified heatwave region and duration. Compared with the active regions of submesoscales in this season, it is found that the two have good spatial consistency [cf. Fig. 1 of Su *et al.* (*26*) and Fig. 2 of Archer *et al.* (*27*)], potentially suggesting that submesoscales may contribute to the heat budget of MHWs.

**Fig. 1.**
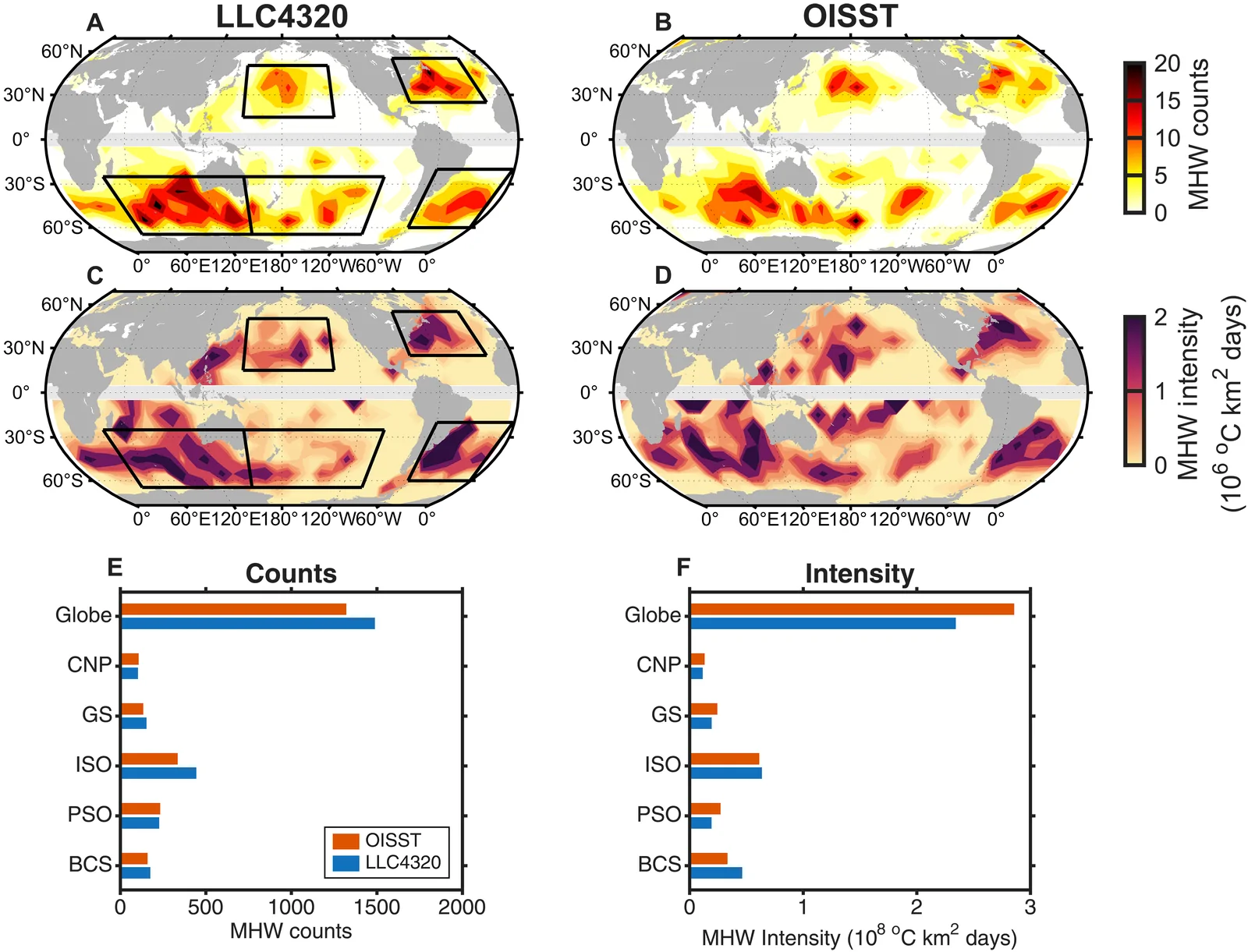
A comparison of MHW events in the winter of 2012. The spatial distributions of (**A** and **B**) the counts and (**C** and **D**) intensity of MHWs over the globe on 10°×10° grids. Each MHW event is assigned to a grid box based on the geometric center of its boundary, and the cumulative intensity is defined as the sum of event-based integrated SST anomalies over space and time assigned to that grid box. (A) and (C) are derived from the LLC4320, while (B) and (D) are from OISST. (**E**) and (**F**) show a comparison of the total counts and intensity integrated over the globe and the five regions denoted as the black boxes in (A) and (C). CNP: Central North Pacific, 150°E-140°W, 25°N-45°N; GS: Gulf Stream, 80°W-20°W, 25°N-55°N; ISO: Indian Ocean Sector of the Southern Ocean, 40°E-150°E, 25°S-65°S; PSO: Pacific Ocean Sector of the Southern Ocean, 150°E-100°W, 25°S-65°S; BCS: Brazil Current System, 60°W-0°W, 20°S-60°S.

**Fig. 2.**
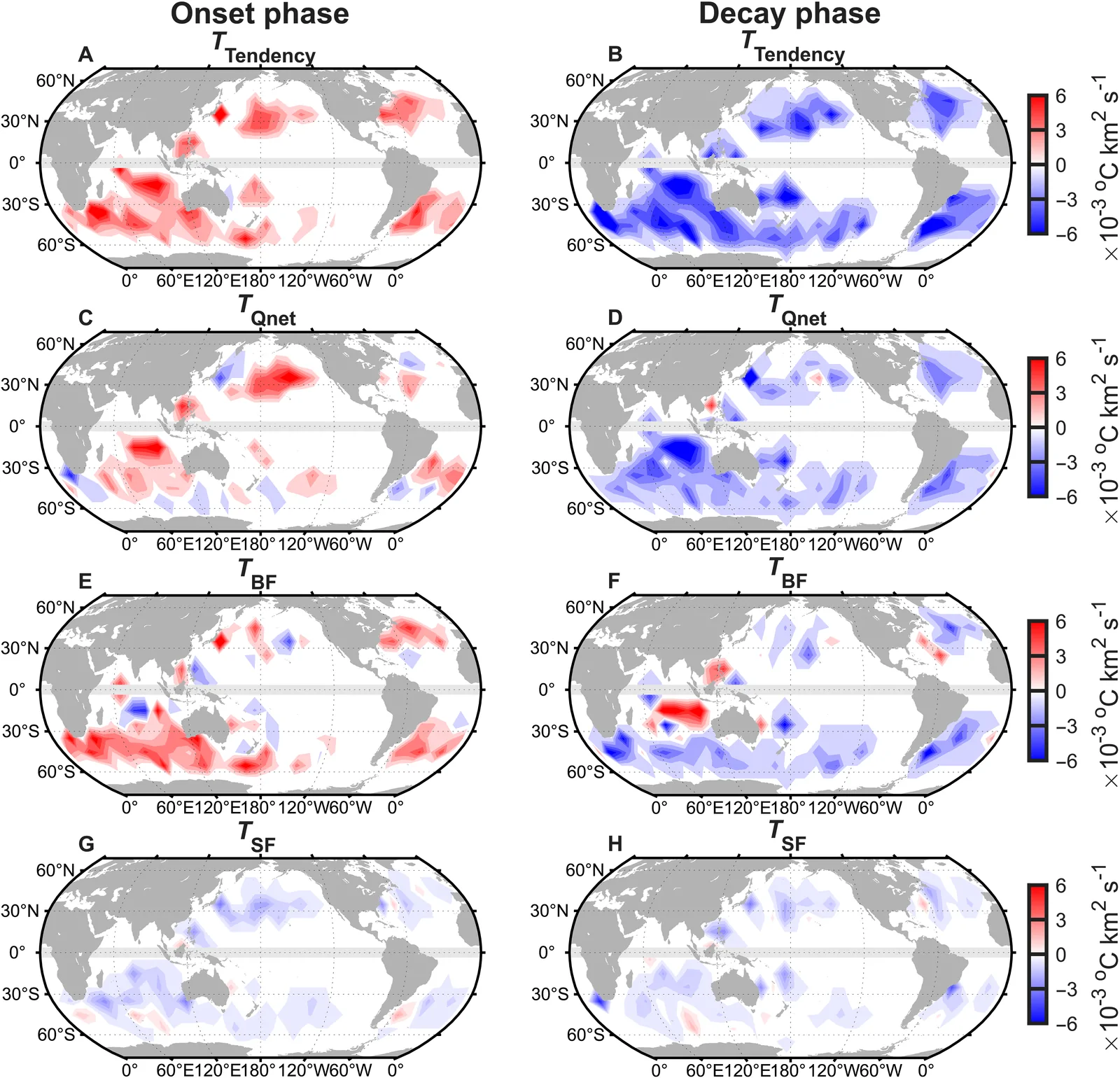
The MHW anomaly heat budget during the onset and decay phases. The spatial distribution of the phase-averaged budget terms: (**A** and **B**) tendency (*T*_Tendency_), (**C** and **D**) sea surface anomalous net heat flux (*T*_Qnet_ with positive sign indicating heating the ocean), (**E** and **F**) the anomalous heat convergence by background currents (*T*_BF_), and (**G** and **H**) the anomalous heat convergence by submesoscale currents (*T*_SF_) over the globe on 10°×10° grids. The left columns show the results during the onset phase, while the right ones for the decay phase.

To further evaluate the LLC4320’s ability to reproduce MHWs, we conducted statistical analyses of the number and intensity over the globe and five subregions: Central North Pacific (CNP), Gulf Stream (GS), Indian Ocean sector of the Southern Ocean (ISO), Pacific Ocean sector of the Southern Ocean (PSO), and Brazil Current System (BCS) (black rectangles in Fig. 1, A and C). The numbers of MHWs simulated by LLC4320 are highly consistent with the observational data, both globally and in the five subregions, with the model slightly overestimating the total number of heatwaves. The model-derived number over the globe is 12.6% higher than the observational number. However, the LLC4320 underestimates heatwave intensity globally. On a global scale, the underestimation of the total heatwave intensity from the model is 18.0%. Nevertheless, for the five key subregions of focus, the total intensity is very close to the observed one (with a bias of only 0.4%). Given that these results are based on 3 months of data, the comparison indicates the capability of the LLC4320 simulation in statistically capturing the heatwave characteristics during this period.

### Submesoscale contribution to the MHW heat budget globally

Following Bian *et al.* (*21*), we focus on the upper 50-m-depth layer to diagnose the heat budget of the SST-derived MHWs. The results of Bian *et al.* (*21*) indicate that the statistical characteristics of heatwaves derived from the depth-averaged temperature in the upper 50 m are consistent with those from SST. This reveals that the upper-ocean heatwaves have a vertical structure, with consistent variation characteristics within the upper 50-m depth (*47*) (this consistency is speculated to be more pronounced in winter due to a deeper mixed layer). On the other hand, the main reason for the choice of a fixed depth instead of the entire mixed layer is based on biological considerations (*21*). Globally, the euphotic zone depth ranges from tens of meters to ∼200 m, but in mid-latitudes, its depth is around 50 m (*48*). The mid-latitude regions are where MHWs and submesoscales are primarily concentrated during the focused period here.

On the basis of the heat budget equation of the upper-50-m averaged temperature anomaly (see Materials and Methods), we calculated the phase-averaged (onset and decay phases, respectively) magnitude of the budget terms for each heatwave, including the tendency term, *T*_Tendency_, the net surface heat flux term *T*_Qnet_, the background-current-induced heat flux term *T*_BF_, and the submesoscale-induced heat flux term *T*_SF_. The residual term is not shown here, because its spatial distribution should be interpreted with caution, as it reflects both unresolved physical processes and numerical uncertainties (see Materials and Methods). The globally averaged magnitude of the residual term corresponds to approximately 12.3% (11.0%) of the *T*_Tendency_ magnitude during the onset (decay) phase, suggesting that the results here capture the dominant evolution of MHWs. The equations (*4*–*6*) here describe the budget of the horizontally integrated temperature anomaly within the heatwave region divided among these contributions. To better illustrate the contributions of different processes, the MHW life cycle is divided into two phases, onset and decay, using the maximum value of the integrated temperature anomaly over the MHW region as the demarcation point in time. We then computed the average magnitude of each budget term during these two phases (Fig. 2). During the onset phase, both *T*_Qnet_ and *T*_BF_ are generally positive, indicating that both terms contribute to the growth of heatwaves. In the decay phase, however, both terms become negative, jointly promoting the decay of heatwaves. Although there are certain regional differences in their relative contributions, this phase-synchronized transition suggests that both air-sea heat flux and heat convergence driven by background currents are key factors governing MHW evolution. The findings are generally consistent with previous long-term studies (*21*), despite focusing only on MHWs in the 2012 winter here.

What distinguishes this study from previous works (*21*) is that the heat budget equation also reveals the contribution from submesoscale processes. Compared with *T*_Qnet_ and *T*_BF_, the *T*_SF_ contribution is smaller in magnitude, yet it does not undergo a sign change during the two phases of the heatwave, i.e., it consistently exerts a cooling contribution to the MHW. Specifically, during the onset phase, it inhibits the intensification of heatwaves, while it accelerates the decay in the decay phase. This characteristic, which differs from the other two processes, potentially indicates that *T*_SF_ is not the primary trigger of heatwave onset, but rather an active feedback arising from the response of submesoscale processes to heatwaves.

Globally, during the MHW onset phase, *T*_BF_ is the largest contributing factor to the MHW growth, while *T*_Qnet_ also makes a considerable contribution with a magnitude slightly smaller. The sum of the two terms largely accounts for the global MHW growth. It is noteworthy that, in contrast, the submesoscale *T*_SF_ tends to inhibit heatwave intensification. Quantitatively, *T*_SF_ reduces the growth rate of MHWs by 6.7% (bootstrap-based 95% confidence interval of [2.6 to 10.4%]), defined as the percentage of the phase-averaged *T*_SF_ relative to the phase-averaged growth rate without *T*_SF_ (i.e., $\frac{|T_{\text{SF}}|}{|T_{\text{Tendency}}|+|T_{\text{SF}}|}\times 100\%$). During the MHW decay phase, *T*_Qnet_ becomes the strongest driver, and *T*_SF_ is also a factor contributing to the heatwave decay, increasing the decay rate by 7.8% [3.4 to 12.8%] (defined as the percentage of the phase-averaged *T*_SF_ relative to the phase-averaged decay rate without *T*_SF_, i.e.,$\frac{|T_{\text{SF}}|}{|T_{\text{Tendency}}|-|T_{\text{SF}}|}\times 100\%$).

### Submesoscale contribution to different MHW types

From a global perspective, the contribution from submesoscales is limited. However, the following analysis will show that the significance of the submesoscale contribution is dependent on MHW types.

Here, the MHWs are divided into four types based on the dominant drivers (see Materials and Methods): *T*_BF_ drives both the intensification and decay (BF&BF type), *T*_BF_ drives the intensification while *T*_Qnet_ drives the decay (BF&Qnet type), *T*_Qnet_ drives the intensification while *T*_BF_ drives the decay (Qnet&BF type), and *T*_Qnet_ drives both the intensification and decay (Qnet&Qnet type). The heat budget terms for these four types are shown in Fig. 3.

**Fig. 3.**
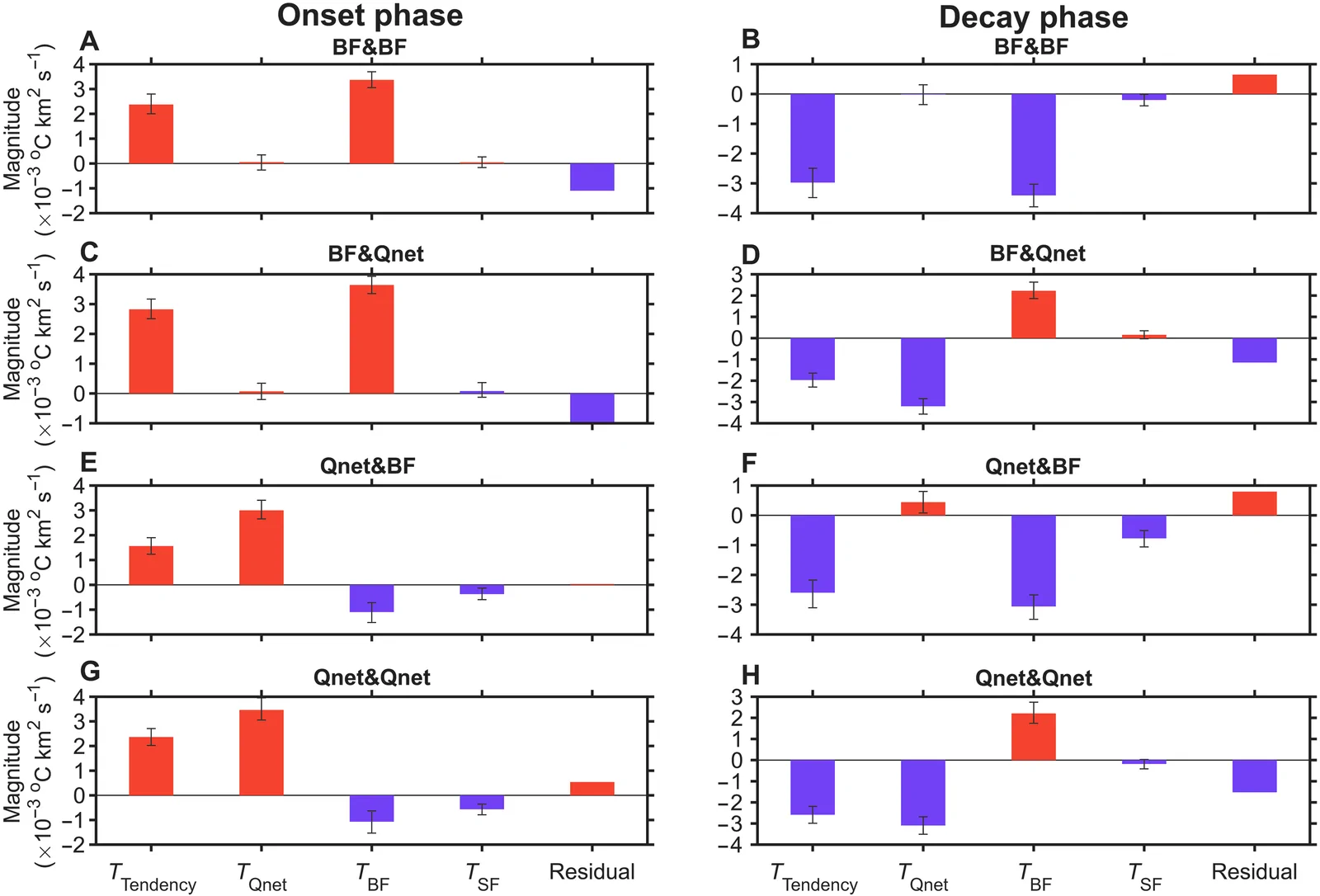
The averaged magnitudes of the MHW budget terms during the different phases for different MHW types. (**A** and **B**) BF&BF, (**C** and **D**) BF&Qnet, (**E** and **F**) Qnet&BF, and (**G** and **H**) Qnet&Qnet. The left columns show the results during the onset phase, while the right ones for the decay phase. The error bars denote 95% confidence intervals of the mean budget terms estimated from event-based samples with 5000 bootstrap resamples. BF&BF: *T*_BF_ drives both the intensification and decay; BF&Qnet: *T*_BF_ drives the intensification, while *T*_Qnet_ drives the decay; Qnet&BF: *T*_Qnet_ drives the intensification while *T*_BF_ drives the decay; Qnet&Qnet: *T*_Qnet_ drives both the intensification and decay. The residual term represents the combined effect of unresolved physical processes and uncertainties associated with the offline diagnostic calculation.

For the BF&BF and BF&Qnet types, *T*_BF_ dominates the intensification of MHWs. However, during the decay phase, *T*_BF_ dominates the decay for the BF&BF type, but tends to inhibit the heatwave decay driven by *T*_Qnet_. *T*_SF_ is always negligible during different phases for the two types. By contrast, *T*_Qnet_ and *T*_BF_ always tend to compete with each other for the Qnet&BF and Qnet&Qnet types. During the onset phase, *T*_Qnet_ dominates the intensification, while *T*_BF_ tends to inhibit it. However, during the decay phase, the other term tends to inhibit the decay, no matter whether *T*_Qnet_ or *T*_BF_ dominates. It is noteworthy that *T*_SF_ becomes non-negligible for these two types, which always makes a negative contribution—it inhibits the heatwave intensification and promotes the heatwave decay. Quantitatively, *T*_SF_ weakens the growth rate by 18.1% [9.3 to 25.1%] in the growing phase for the Qnet&BF and Qnet&Qnet types (hereinafter referred to as Qnet-driven). During the decay phase, *T*_SF_ increases the decay rate by 21.7% [8.8 to 43.9%] on average. The results above indicate that for specific types of heatwaves, submesoscale processes reduce MHW severity by suppressing intensification and accelerating decay. Despite the non-negligible residual term, it does not alter our main conclusion because the diagnosed submesoscale contribution is evaluated primarily from the comparison between *T*_SF_ and *T*_Tendency_.

### Weakening of submesoscale activity during MHWs

On the basis of the above analysis, it can be concluded that the contributions of submesoscale processes to the evolution of the Qnet-driven MHWs are non-negligible. Figure 4 presents the global spatial distribution of the heat flux induced by submesoscale processes, i.e., *T*_SF_, during the occurrence of Qnet-driven MHWs (including both the onset and decay phases). When only Qnet-driven MHWs are considered, the submesoscale heat flux (*T*_SF_) is more pronounced (Fig. 4A), being stronger than the results obtained from the analysis of all MHW types (Fig. 2, G and H). According to Eq. 6, *T*_SF_ can be decomposed into four components: the submesoscale-background coupled horizontal flux term (*T*_SBHF_), the submesoscale-background coupled vertical flux term (*T*_SBVF_), as well as the horizontal (*T*_SHF_) and vertical (*T*_SVF_) flux terms driven solely by submesoscale processes.

**Fig. 4.**
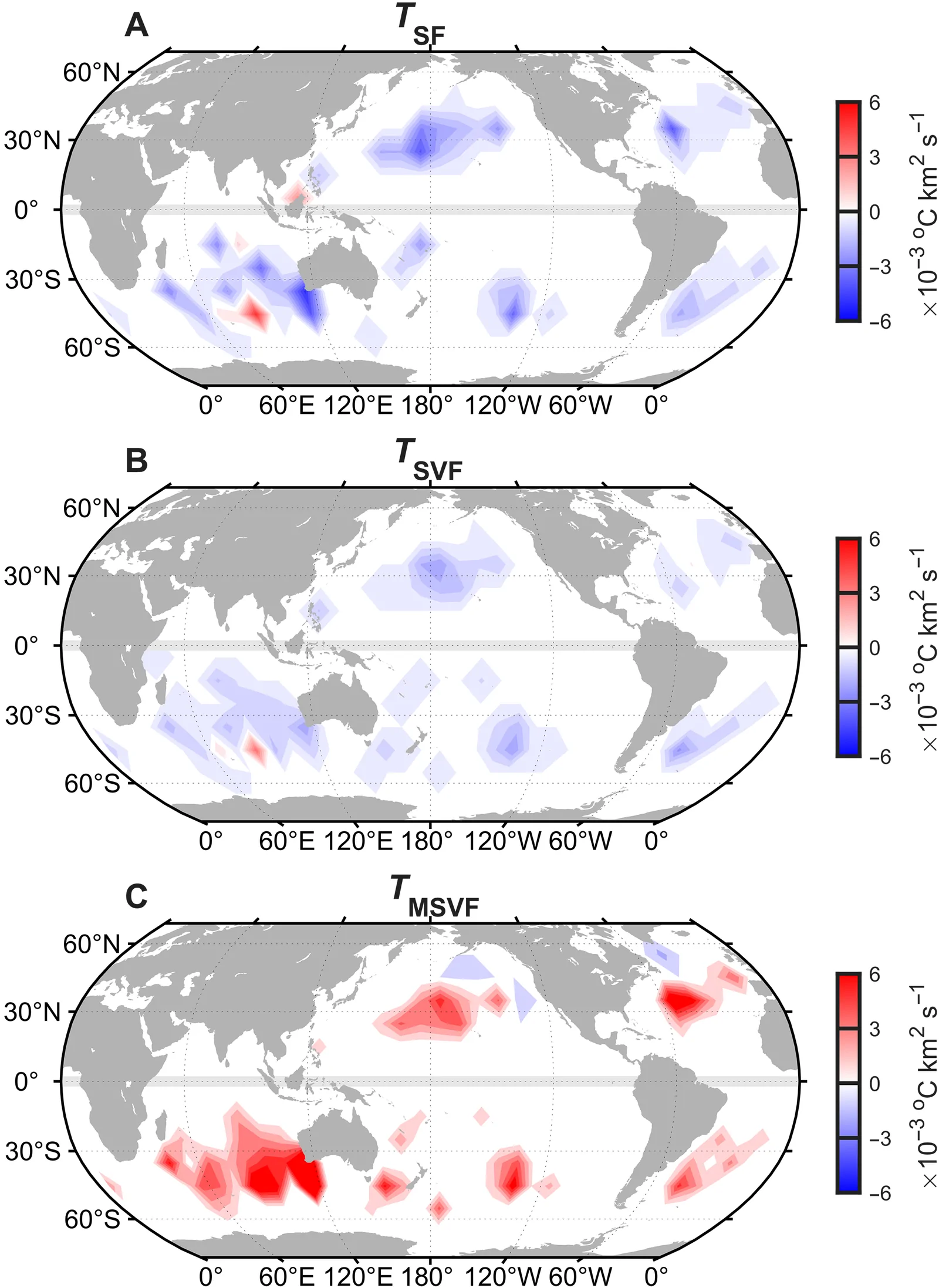
Integrated submesoscale-induced heat fluxes during Qnet-dominated MHWs. (**A**) Submesoscale heat flux, *T*_SF_; (**B**) Submesoscale vertical flux, *T*_SVF_; and (**C**) Mean submesoscale vertical flux, *T*_MSVF_. The anomalies are also averaged on 10°×10° grids over the globe.

However, our calculations indicate that the vertical flux driven solely by submesoscale processes (*T*_SVF_; hereinafter, it is directly referred to as submesoscale-induced vertical heat flux for convenience) dominates the total flux term (*T*_SF_), accounting for 68.3% [58.9 to 84.8%] of the globally averaged magnitude of *T*_SF_ (Fig. 4B). Therefore, this study focuses primarily on the mechanisms underlying the contribution of *T*_SVF_ during heatwave events. It should be noted that a 60-day moving average is applied to remove the influence of background seasonal variations on the heat budget. Studies have shown that *T*_SVF_ under spatiotemporal averaging is always upward (*23*, *32*, *43*), which is linked dynamically to the extraction of potential energy by submesoscales (*36*). If we examine the background vertical heat flux with a 60-day moving average during the occurrence of MHWs, it also yields an upward vertical heat flux that is consistent with the results reported in previous studies (Fig. 4C), but the vertical heat flux is weaker during MHWs. The negative *T*_SVF_ contribution is mainly a result of a reduction of the upward vertical heat flux during MHWs. During the occurrence of MHWs, *T*_SVF_ accounts for 19.4% [17.1 to 21.9%] relative to *T*_MSVF_, which indicates that the submesoscale vertical heat flux is weakened by 19.4% when MHWs occur. This is inevitably related to weakened activity of submesoscales during MHWs (*32*, *33*).

To verify this, we calculated the submesoscale activity anomaly during MHWs (see Materials and Methods). The results indicate that submesoscale activity is weakened by 8.8% [7.3 to 10.4%] in regions where MHWs occur (fig. S3A). Previous studies have shown that submesoscale intensity is related to multiple factors, among which the mixed-layer depth squared is one of the key influencing factors (*36*). It has been reported that the mixed layer depth shoals by ∼8.1% during heatwaves (*49*), which, in turn, leads to a reduction in the storage of available potential energy within the mixed layer and smaller, slower submesoscale instabilities (*50*), thereby decreasing the submesoscale rate of potential energy extraction and intensity.

To verify whether the mixed layer becomes shallower, we calculated the mixed layer depth anomalies during MHWs. The results show that the mixed layer depth exhibits a negative anomaly during heatwaves, signifying a shoaling mixed layer by 9.3% [6.1 to 12.1%] relative to the mean mixed layer depth (fig. S3, B and D). From a dynamical perspective, this change is fairly intuitive. As a driving factor, *T*_Qnet_ always exerts a positive contribution during Qnet-driven MHWs. In the process of heating the ocean, it inevitably increases the oceanic vertical temperature gradient, leading to shoaling of the mixed layer depth (*49*).

### Weakening of submesoscale vertical heat flux in GLORYS

Given that the results are derived solely from the winter 2012 dataset, to further verify the robustness of these findings, we also investigated the seasonal variations in the submesoscale vertical heat flux during the occurrence of wintertime MHWs using long-term GLORYS reanalysis data and a parameterization method (*36*, *51*) (see Materials and Methods). No distinction is made between different types of MHWs in this analysis. Nevertheless, a reduction of the submesoscale vertical heat flux is consistently evident in the reanalysis data during MHWs (fig. S4, A, B). Globally, the submesoscale vertical heat flux is decreased by 8.7% [7.5 to 10.2%], which is in close agreement with the results directly diagnosed from the LLC4320 dataset.

To distinguish the contributions of mixed layer depth and buoyancy gradients, we applied a 60-day moving average to the mixed layer depth and the buoyancy gradient separately (i.e., removing variations in the corresponding variable during MHW while retaining variations in the other variable), and recalculated the submesoscale vertical heat flux. The results show that the weakening of the submesoscale vertical heat flux is dominated by changes in the mixed-layer depth (fig. S4, C and D), further supporting our conclusion above.

## DISCUSSION

The results here reveal that the reduction in the intensity of the vertical heat flux induced by submesoscale processes can suppress the severity of MHWs that are driven by the air-sea heat flux (Fig. 5). Further analytical results indicate that this is mainly attributed to the fact that, when MHWs occur, anomalies in the net air-sea heat flux strengthen the stratification and reduce the depth of the mixed layer. This leads to a decrease in available potential energy, which inhibits the activity of submesoscale processes. Consequently, the vertical heat flux induced by submesoscale processes is attenuated, thereby suppressing the intensification and accelerating the attenuation of MHWs. In contrast to the net atmospheric heat input, the submesoscale-induced heat flux primarily redistributes heat within the mixed layer. It can be regarded as a submesoscale negative feedback effect on MHWs. The findings demonstrate that submesoscale processes exert a non-negligible inhibitory effect on the severity of MHWs.

**Fig. 5.**
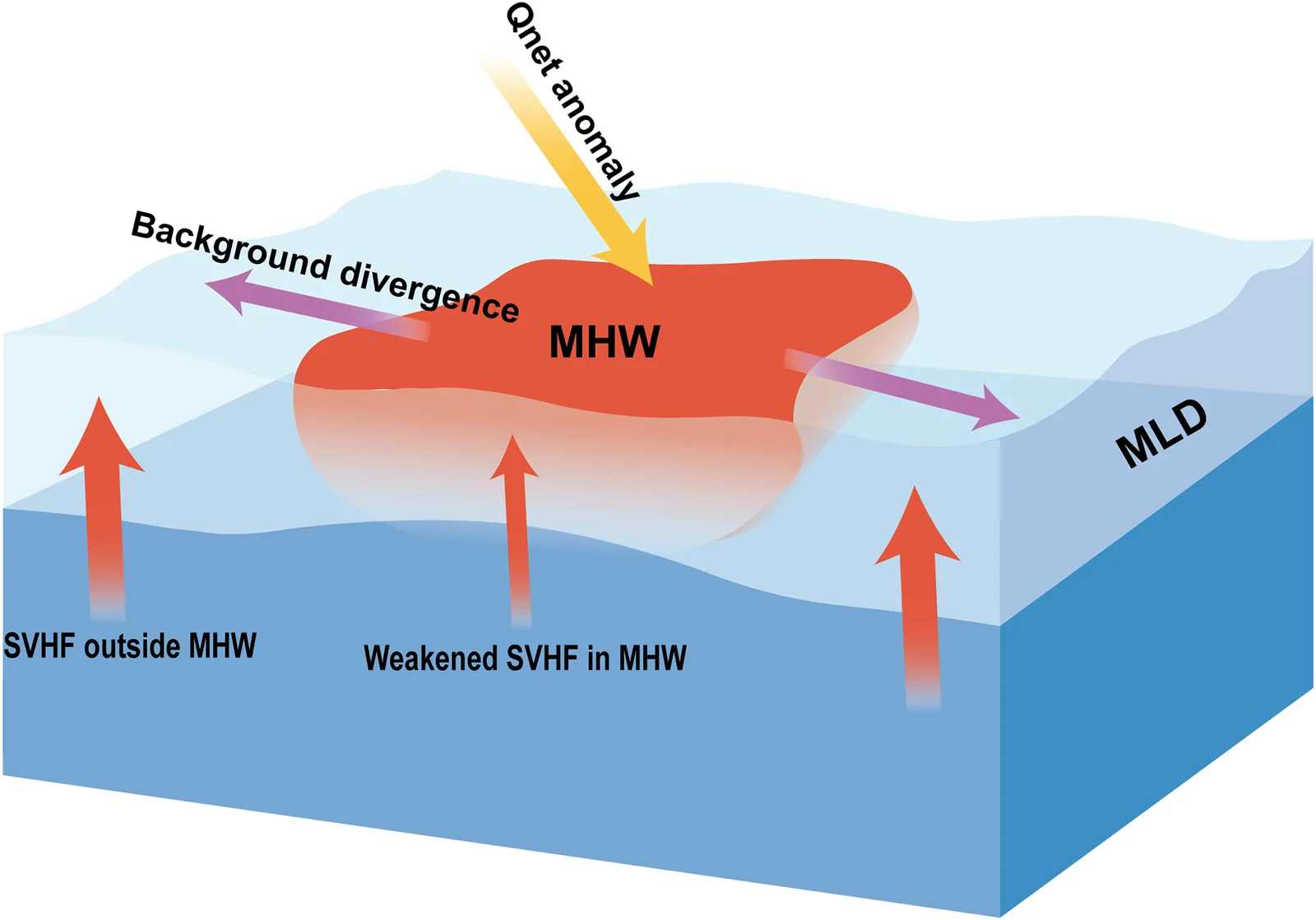
A diagram of the submesoscale negative feedback mechanism regulating MHW severity in a surface-flux-induced event. When the positive net surface heat flux (Qnet; yellow arrow) drives MHWs, the vertical stratification is also strengthened by the anomalous heat flux, leading to a shallower mixed layer. A shallower mixed layer reduces the release of available potential energy, which weakens the activity of submesoscale processes compared to the regions outside the MHW and hence weakens the submesoscale vertical heat flux (SVHF; red arrow). This subsequently exerts a negative feedback on the heat budget of the heatwave and suppresses its severity. During this process, the background circulation (pink arrow) also tends to diverge the heat accumulated in the heatwave. The figure was created entirely by the authors using Adobe Illustrator.

Limitations of the present work should be noted. Because the heat-budget terms are diagnosed offline rather than directly output by the model, the diagnosed budget inevitably contains uncertainties. In addition, the lack of mixing-related outputs prevents a direct quantification of horizontal and vertical mixing contributions, which are therefore included in the residual term. Moreover, because the analysis is restricted to MHWs whose onset and termination both occur within the 3-month winter window, long-duration and cross-seasonal events are excluded, and the conclusions may not directly apply to such events.

This study highlights the direct contribution of submesoscale processes as a negative feedback on MHWs that are driven by sea surface heat flux, which may not necessarily apply to background-current-driven ones. Furthermore, the indirect impacts of submesoscale processes on MHWs should also be taken into account. Here, the direct contribution of submesoscale processes to background-current-driven MHWs is negligible. However, submesoscale processes can affect the intensity of mesoscale flow fields through inverse energy cascade, which indirectly influences the heat convergence induced by mesoscale flow fields (*50*, *52*, *53*). On the other hand, considering the restratification effect of submesoscale processes, in addition to directly regulating MHW severity via vertical heat fluxes, submesoscale processes may also indirectly affect heatwave severity by influencing mixing intensity (*54*, *55*). These represent important directions for future research.

## MATERIALS AND METHODS

### Submesoscale-permitting model simulation

To conduct the diagnosis of the heat budget of upper-ocean heatwaves, we used data from LLC4320, including temperature and three-dimensional current field in the upper 50 m of the ocean. LLC4320 is a simulation of the Massachusetts Institute of Technology general circulation model (MITgcm) configured on a latitude-longitude polar cap (LLC) grid. With its horizontal resolution at 1/48° and with the realistic tidal forcings, the output of LLC4320 has been used for many purposes, including to explore the submesoscale dynamics in different parts of the World Ocean (*56*, *57*), to compare internal gravity wave SSH spectra against globally available moored measurements (*58*), and to design calibration and validation approaches for SWOT-measured SSH signals (*59*). It has been evaluated against satellites (*60*), although it does not assimilate ocean observations. The model has a spatial resolution of 1/48° and 90 vertical layers, which can partially resolve submesoscale processes in the upper ocean, especially in winter (*61*).

The model was successively initialized from a series of simulations with resolutions of 1/6°, 1/12°, and 1/24°. The K-profile parameterization (KPP) scheme was adopted for the simulation. Atmospheric forcing driving the simulation was obtained from the European Centre for Medium-Range Weather Forecasts (ECMWF), with a temporal resolution of 6 hours and a spatial resolution of 0.14°. For the calculation of sea surface net heat flux, we directly used the sea surface fluxes output from the simulation.

LLC4320 was run for a 14-month simulation period, from September 2011 to November 2012. The original temporal resolution of the model output is 1 hour, while the data used in this study have been daily-averaged.

This model output has been validated through comparisons with observations in different marine regions (*52*, *56*, *57*, *60*). These results indicate that despite certain deviations from observations, the model output is consistent with observations in terms of the statistical characteristics of dynamical environmental parameters. Regarding the upper-ocean temperature focused here, several studies have also evaluated its consistency with observations. Validation results by Gallmeier *et al.* (*60*) show that LLC4320 reproduces the observed distribution of SST patterns well both globally and regionally (here, SST from LLC4320 is the uppermost 0.5-m layer of the simulation). On the basis of high-resolution remote sensing data, Dong *et al.* (*54*) used the first-order structure function to evaluate SST spatial variability, and their results demonstrate that the statistical characteristics of the SST variability simulated by the model above its effective resolution are basically consistent with observations. Other submesoscale-permitting simulations at similar resolutions have many similar upper ocean features and similar vertical buoyancy flux rates (*62*). These validation results indicate that the model performs well in simulating temperature variability. However, it should be noted that there is a systematic bias in the SST simulated by LLC4320 compared with observations (*60*), and this bias has been removed on the basis of observations before the MHW detection (see MHW identification).

### Sea surface temperature

To verify the model’s ability to simulate heatwaves, we used the Optimum Interpolation SST data provided by the National Oceanic and Atmospheric Administration (NOAA OISST V2.1). By merging observations from various platforms, such as satellites, buoys, and Argo floats, the dataset has a spatial resolution of 0.25° × 0.25° and a temporal resolution of 1 day (*63*, *64*). Data with a temporal coverage from 1982 to 2012 were used in this work. These data have been widely used in MHW research (*4*, *15*, *65*).

### Reanalysis data

The ocean data provided by Global Ocean Physics Reanalysis (GLORYS12V1) were also used in this work. GLORYS12V1 is simulated on the basis of the NEMO platform forced by ECMWF ERA-Interim and ERA5 reanalysis for recent years. Observations are assimilated in the simulation, including along-track altimeter data, Satellite SST, sea ice concentration, and in situ vertical profiles of temperature and salinity. The spatial resolution of the data is 1/12° with 50 vertical layers. In this work, the daily temperature and salinity in the upper 50 m from 1995 to 2020 were used for the diagnosis of submesoscale-induced vertical flux (see the “Parameterization of submesoscale-induced vertical heat flux” section).

### MHW identification

The definition of MHWs in this study follows the definition proposed by Hobday *et al.* (*2*), i.e., a 5-day consecutive anomalous high SST exceeding the 90th percentile of the seasonally varying climatology. For the OISST data, MHWs are identified directly at each grid point. The climatology reference period here spans from 1 January 1982 to 31 August 2011, while the period for MHW identification is consistent with the model’s simulation period, i.e., September 2011 to November 2012. Subsequently, the extent of each MHW is determined on the basis of grid connectivity, which is defined as a single heatwave event. Furthermore, whether two heatwaves at adjacent time steps belong to the same event is determined by the presence of overlapping areas. On the basis of this, the temporal evolution of an MHW event with spatial extent is derived. For spatial binning on 10° × 10° grids, the representative location of each MHW event is defined as the geometric center of its boundary. The intensity of each event is calculated as the SST anomaly integrated over the heatwave area and duration, and is then assigned to the 10° × 10° grid box containing the representative event location.

For heatwave identification, several constraints require specific clarification here:

1) First, a heatwave event is recognized only when five adjacent grid points, corresponding to a heatwave area of over 3000 km^2^ in low latitudes, all experience heatwaves simultaneously. The definition of this threshold is not based on any dynamical reasons; rather, it serves to exclude some small-area heatwaves that are likely sensitive to choices such as spatial averaging.

2) Second, consistent with the first constraint, two heatwaves at adjacent time steps are deemed the same event only if their overlapping area covers more than five grid points.

3) Third, aligned with the definition proposed by Hobday *et al.* (*2*), the heatwaves defined herein must have a duration exceeding 5 days.

4) Last, if an identified heatwave exhibits multiple cycles of intensification and weakening, with the time interval between two adjacent intensity peaks exceeding 5 days, it is subdivided into multiple heatwave events based on the number of such peaks.

For the LLC4320 data, we first conducted spatial averaging to get SST data with a resolution consistent with OISST. Subsequently, we applied the same method to identify MHWs. The key difference is that, given the model only provides 1 year of data, we relied on observational climatological temperature data as a reference to identify heatwaves.

As noted earlier, the LLC4320 model simulates SST variation in good agreement with observations but exhibits a systematic bias. In light of this, we calibrated the LLC4320 SST against observations as follows. First, we separately computed 60-day moving averages of SST at each grid point for both the simulation and observation during the study period. This processing removed anomalous SST variability and yielded time series that primarily reflect seasonal variation. On the basis of the bias between these two time series, we calibrated the unsmoothed raw model temperatures by subtracting this bias. For the calibrated data, we then identified heatwaves during the period using the observed climatological SST data as a reference. It should be noted that heatwave budgets focus on short-term abrupt changes in temperature; thus, this calibration does not affect subsequent analyses of heat budgets.

To visually demonstrate the effectiveness of the MHW identification after calibration, we compared the identified MHWs with observations using a time series at a selected location in the central Pacific Ocean (fig. S1). It can be observed that heatwave events identified from the LLC4320 SST are highly consistent with the observations both in terms of duration and intensity, despite the model’s inability to fully and accurately replicate the observed SST curve over time.

Furthermore, considering that this study focuses only on winter seasons when submesoscales are relatively active (January, February, and March in the Northern Hemisphere; July, August, and September in the Southern Hemisphere), we filtered MHWs to retain only those whose onset and end times both fall within this period. This criterion focuses the analysis on MHWs that develop and decay within the winter season, but it also excludes long-duration or cross-seasonal MHW events whose onset or termination occurs outside this winter window.

Statistical analyses of grid-point MHWs show that LLC4320 exhibits strong agreement with observations in terms of heatwave count, duration, average intensity, and cumulative intensity, both in spatial distribution and in global and regional statistical results (our statistical results indicate that even when comparing MHWs over the whole year, the statistical characteristics derived from the two datasets remain highly similar). This demonstrates that the model performs well in reproducing various MHW metrics (fig. S2).

### Heat budget of MHWs

For an MHW event, the heat budget of the upper 50 m corresponding to its coverage area can be characterized by the following equation

$$\frac{\partial }{\partial t}\iint_{\text{MHW}}\langle T\rangle \mathrm{dA}=\iint_{\text{MHW}}\langle -\nabla \left(uT\right)\rangle \mathrm{dA}+\iint_{\text{MHW}}\frac{Q_{\mathrm{net}}}{\rho_{0}C_{p}h}\mathrm{dA}+\text{Res}$$ (1)

Here, *T* is the temperature (°C), **u** = (*u*, *v*, *w*) is the three-dimensional current field (m s^−1^), *Q*_net_ is the net sea surface heat flux excluding the solar radiation at 50-m depth (W m^−2^), $\rho_{0}$ = 1024 kg m^−3^ is the seawater density, *C*_p_ = 3800 J kg^−1^°C^−1^ is the seawater heat capacity, and *h* = 50 m is the upper layer thickness for analysis. The left-hand side of the equation represents the temporal tendency term of horizontally integrated temperature anomaly during the heatwave (*T*_Tendency_). On the right-hand side, the first term is the heat flux induced by the current field, the second term is the net heat flux term (*T*_Qnet_). The angle brackets ⟨⟩ denote the depth average over the upper 50 m.

The last term is the residual term, which includes heat fluxes due to horizontal and vertical mixing (which cannot be diagnosed here due to limitations in the model output data) as well as computational biases that may arise from offline diagnostics. The residual term is not necessarily negligible, since mixing processes, particularly vertical mixing, can make a non-negligible contribution to energetic current systems (*21*). Nevertheless, the main conclusion is expected to be insensitive to such diagnostic uncertainties because it relies primarily on the comparison between the submesoscale-induced heat flux and the tendency terms.

To examine the contribution of submesoscale processes, we first perform a Reynolds decomposition on the original current and temperature fields, decomposing them into a background component and a submesoscale component, i.e.,

$$\left\{\begin{array}{c} u=\bar{u}+u^{\prime }\\ T=\bar{T}+T^{\prime } \end{array}\right.$$ (2)

Following the work of Su *et al.* (*26*), we define signals with a spatial scale smaller than 0.5° as submesoscale processes. Specifically, for LLC4320 data at its original resolution, mesoscale signals at each grid point are defined as the average over a 0.5° × 0.5° region centered at that grid point, while submesoscale processes correspond to the anomalies relative to this mean.

After obtaining the mesoscale and submesoscale signals, the heat convergence effect induced by the flow field can be decomposed into

$$-\nabla \left(uT\right)=-\nabla \left(\bar{u}\bar{T}\right)-\nabla \left(\bar{u}T^{\prime }\right)-\nabla \left(u^{\prime }\bar{T}\right)-\nabla \left(u^{\prime }T^{\prime }\right)$$ (3)

The first term on the right-hand side of the equation represents the heat flux induced by the background current field (*T*_BF_), while the subsequent three terms are all considered to be the heat flux driven by submesoscales (*T*_SF_). Combining the above results, the heat budget equation for heatwaves can be expressed as follows,

$$\begin{array}{c} \underset{T_{\text{tendency}}}{\underbrace{\frac{\partial }{\partial t}\iint_{\text{MHW}}\langle T\rangle \mathrm{dA}}}=\\ \underset{T_{\text{BF}}}{\underbrace{\iint_{\text{MHW}}\langle -\nabla \left(\bar{u}\bar{T}\right)\rangle \mathrm{dA}}}+\underset{T_{\text{SF}}}{\underbrace{\iint_{\text{MHW}}\langle -\nabla \left(\bar{u}T^{\prime }\right)-\nabla \left(u^{\prime }\bar{T}\right)-\nabla \left(u^{\prime }T^{\prime }\right)\rangle \mathrm{dA}}}+\underset{T_{\text{Qnet}}}{\underbrace{\iint_{\text{MHW}}\frac{Q_{\text{net}}}{\rho_{0}C_{p}h}\mathrm{dA}}}+\text{Res} \end{array}$$ (4)

Before calculating the heat budget, each involved variable is first vertically averaged, then processed into data consistent with the OISST resolution via regional averaging, and subsequently integrated over the heatwave area.

For the submesoscale heat flux term (*T*_SF_), it can be further decomposed into horizontal and vertical contributions, expressed as follows

$$\begin{array}{c} T_{\text{SF}}=\iint_{\text{MHW}}\langle -\nabla \left({\bar{u}}_{h}T^{\prime }\right)-\nabla \left(u_{h}^{\prime }\bar{T}\right)\rangle \mathrm{dA}+\\ \iint_{\text{MHW}}\langle -\nabla \left(u_{h}^{\prime }T^{\prime }\right)\rangle \mathrm{dA}+\iint_{\text{MHW}}\langle -\frac{\partial \left(\bar{w}T^{\prime }+w^{\prime }\bar{T}\right)}{\partial z}\rangle +\iint_{\text{MHW}}\langle -\frac{\partial \left(w^{\prime }T^{\prime }\right)}{\partial z}\rangle \mathrm{dA} \end{array}$$ (5)

Under the rigid-lid approximation, the last two terms on the right-hand side of the equation can be further simplified,

$$\begin{array}{c} T_{\text{SF}}=\underset{T_{\text{SBHF}}}{\underbrace{\iint_{\text{MHW}}\langle -\nabla \left({\bar{u}}_{h}T^{\prime }\right)-\nabla \left(u_{h}^{\prime }\bar{T}\right)\rangle \mathrm{dA}}}+\\ \underset{T_{\text{SHF}}}{\underbrace{\iint_{\text{MHW}}\langle -\nabla \left(u_{h}^{\prime }T^{\prime }\right)\rangle \mathrm{dA}}}+\underset{T_{\text{SBVF}}}{\underbrace{\iint_{\text{MHW}}\frac{{\left(\bar{w}T^{\prime }\right)}_{50\;m}+{\left(w^{\prime }\bar{T}\right)}_{50\;m}}{h}\mathrm{dA}}}+\underset{T_{\text{SVF}}}{\underbrace{\iint_{\text{MHW}}\frac{{\left(w^{\prime }T^{\prime }\right)}_{50\;m}}{h}\mathrm{dA}}} \end{array}$$ (6)

Here, *h* = 50 m. The first and third terms on the right-hand side are the horizontal and vertical heat fluxes induced by the submesoscale-background coupling effect, *T*_SBHF_ and *T*_SBVF_; the second and fourth terms are the horizontal and vertical heat fluxes induced solely by submesoscale perturbations, *T*_SHF_ and *T*_SVF_. Before calculating the heat budget, each term was adjusted by subtracting its 60-day moving average to eliminate the influence of background seasonal variations. In this study, we define background seasonal variations as mean fluxes. Taking the vertical heat flux driven solely by submesoscale processes, as discussed in this paper as an example, the flux calculated on the basis of the 60-day moving averages is defined as the mean flux, *T*_MSVF_. Unless otherwise specified, this flux is directly referred to as submesoscale-induced vertical heat flux in this work.

After these budget terms are diagnosed, they are averaged separately over the onset and decay phases of each MHW, using the time when the horizontally integrated temperature anomaly over the MHW region reaches its maximum as the demarcation point. The MHW type is then determined objectively from the dominant budget term during the onset and decay phases. For each phase, the dominant driver is defined as the one of *T*_Qnet_ and *T*_BF_ that acts in the same direction as *T*_Tendency_ and has the larger phase-averaged magnitude. Each MHW event is then classified according to the dominant drivers during the onset and decay phases, yielding four types: BF&BF, BF&Qnet, Qnet&BF, and Qnet&Qnet.

The phase-averaged budget terms are used to quantify the relative contribution of each process. Specifically, the contribution of submesoscale processes to the growth or decay rate is calculated from the phase-averaged *T*_SF_ relative to the corresponding phase-averaged rate that would occur without *T*_SF_.

### Submesoscale activity

The activity of submesoscale processes is typically characterized by the normalized vertical relative vorticity, one version of the Rossby number,

$$\frac{\xi }{f}=\frac{v_{x}-u_{y}}{f}$$ (7)

Submesoscales are active when the vertical relative vorticity is comparable to the planetary vorticity (*24*, *25*), i.e., $|\frac{\xi }{f}|$ ∼ 1. For the 0.25° resolution grid points, the SD of the normalized vertical relative vorticity, derived from LLC4320 data within a 0.25° × 0.25° domain centered at each grid point, was calculated to characterize the corresponding submesoscale activity. Similarly, the 60-day moving average was subtracted from the submesoscale activity to eliminate the influence of background seasonal variations.

### Parameterization of submesoscale-induced vertical heat flux

Given that the heat budget of heatwaves is primarily driven by the vertical heat flux induced by submesoscale processes, we combined an existing parameterization scheme with the GLORYS12V1 reanalysis data to assess changes in submesoscale-driven vertical heat flux during heatwave events.

The scheme applied here was proposed by Fox-Kemper *et al.* (*36*) (hereinafter F08) based on the theory of mixed-layer baroclinic instability, and the corresponding vertical heat flux can be expressed as,

$$T_{\text{SVF}}=C_{e}\frac{∆S}{L_{f}}\frac{H^{2}\nabla_{h}\bar{b}{\cdot \nabla }_{h}\bar{T}}{|f|}\mu \left(z\right)$$ (8)

Here, *C*_e_ = 0.06 is a constant, *H* is the mixed layer depth [defined as the depth where the density difference reaches 0.03 kg m^−3^ compared to the value at 10 m depth (*66*)], and $\nabla_{h}\bar{b}$ and $\nabla_{h}\bar{T}$ are the background GLORYS-resolved horizontal buoyancy gradient and temperature gradient averaged over the mixed layer, respectively. $\frac{∆S}{L_{f}}$ is a scale factor to make the scheme workable in coarse-resolution models (*51*) [$∆S=\frac{1}{2}\left(∆x+∆y\right)$ is the grid size, $L_{f}=\text{max}\left\{\frac{\mathrm{NH}}{|f|},\frac{{|\nabla_{h}\bar{b}|}^{2}}{f^{2}},500\right\}$ is the frontal scale], and α is the seawater thermal expansion coefficient. μ(*z*) is the vertical structure function and is expressed as,

$$\mu \left(z\right)=\left[1-{\left(\frac{2z}{H}+1\right)}^{2}\right]\left[1+\frac{5}{21}{\left(\frac{2z}{H}+1\right)}^{2}\right]$$ (9)

The F08 scheme has been implemented in different ocean and climate models (*51*, *67*, *68*). On the basis of the GLORYS reanalysis data, we calculated the submesoscale vertical heat fluxes using temperature and salinity data from 1995 to 2020. Furthermore, we identified MHWs for the period 2011–2020 and computed the corresponding submesoscale vertical heat fluxes. It should be noted that before the calculations, we removed the climatological seasonal variation characteristics of the submesoscale vertical heat fluxes.
