# Supplementary material for: Submesoscale feedback suppresses marine heatwaves
Source: Sci Adv. 2026 Oct 21;12(43):eaei4624. doi: 10.1126/sciadv.aei4624 (PMC13685953; doi:10.1126/sciadv.aei4624)
Supplement: Supplementary file 1 [file sciadv.aei4624_sm.pdf]

Science Advances
AAAS

Supplementary Materials for

Submesoscale feedback suppresses marine heatwaves

Jihai Dong *et al.*

Corresponding author: Jihai Dong, jihai_dong@nuist.edu.cn; Zhiyou Jing, jingzhiyou@scsio.ac.cn; Changming Dong, cmdong@nuist.edu.cn

Sci. Adv. **12**, eaei4624 (2026)
DOI: 10.1126/sciadv.aei4624

This PDF file includes:

Figs. S1 to S4

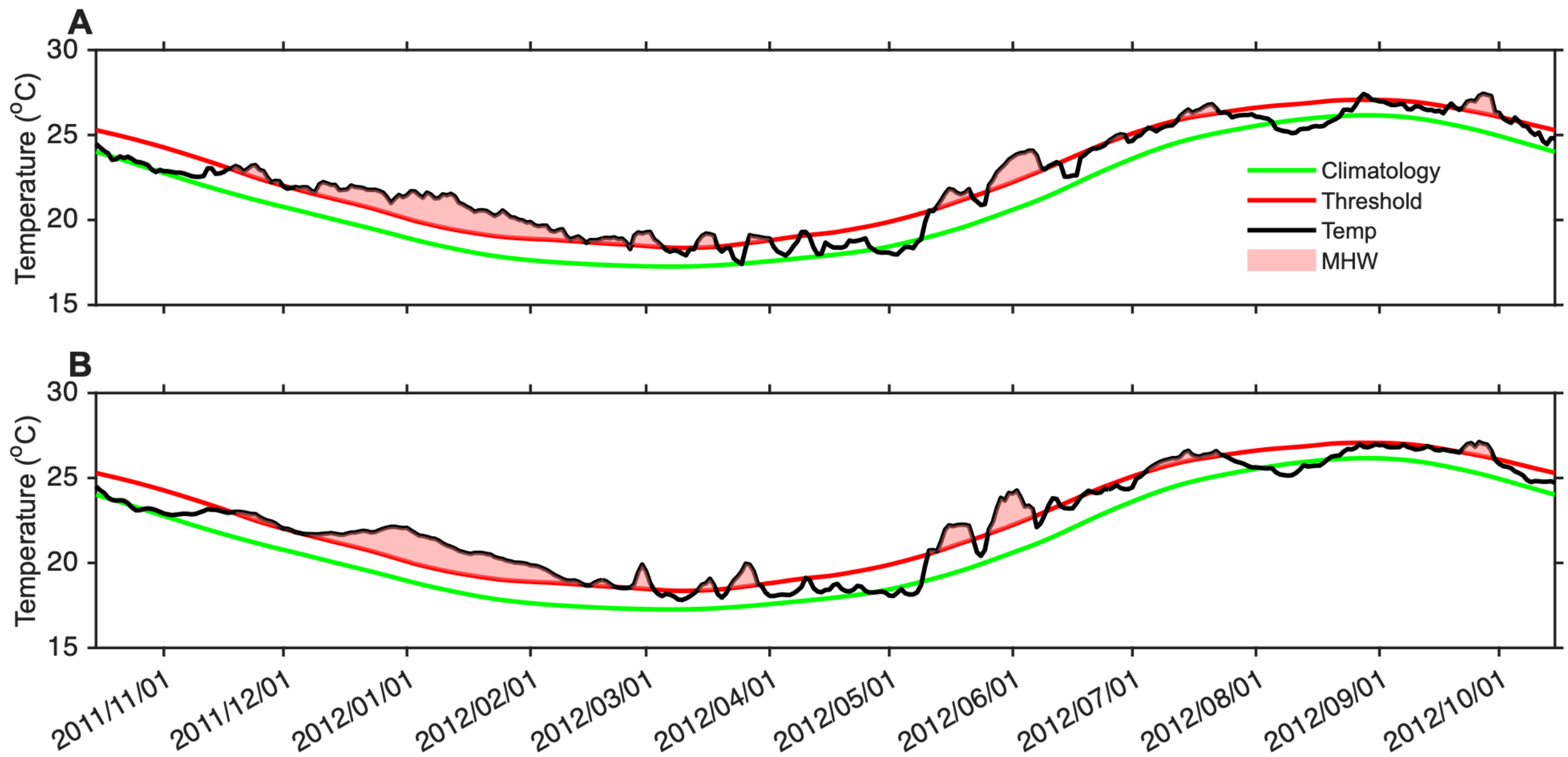

Fig. S1. An example of MHWs identified during the simulation period at a location (175.875°W, 31.375°N) in the North Central Pacific. MHWs identified from the (A) OISST observation and (B) LLC4320 simulation (shaded area). The green line represents the climatological SST, while the red line represents the corresponding 90th percentile as the threshold for determining MHWs. The black curves show the time series of the observed and simulated SST.

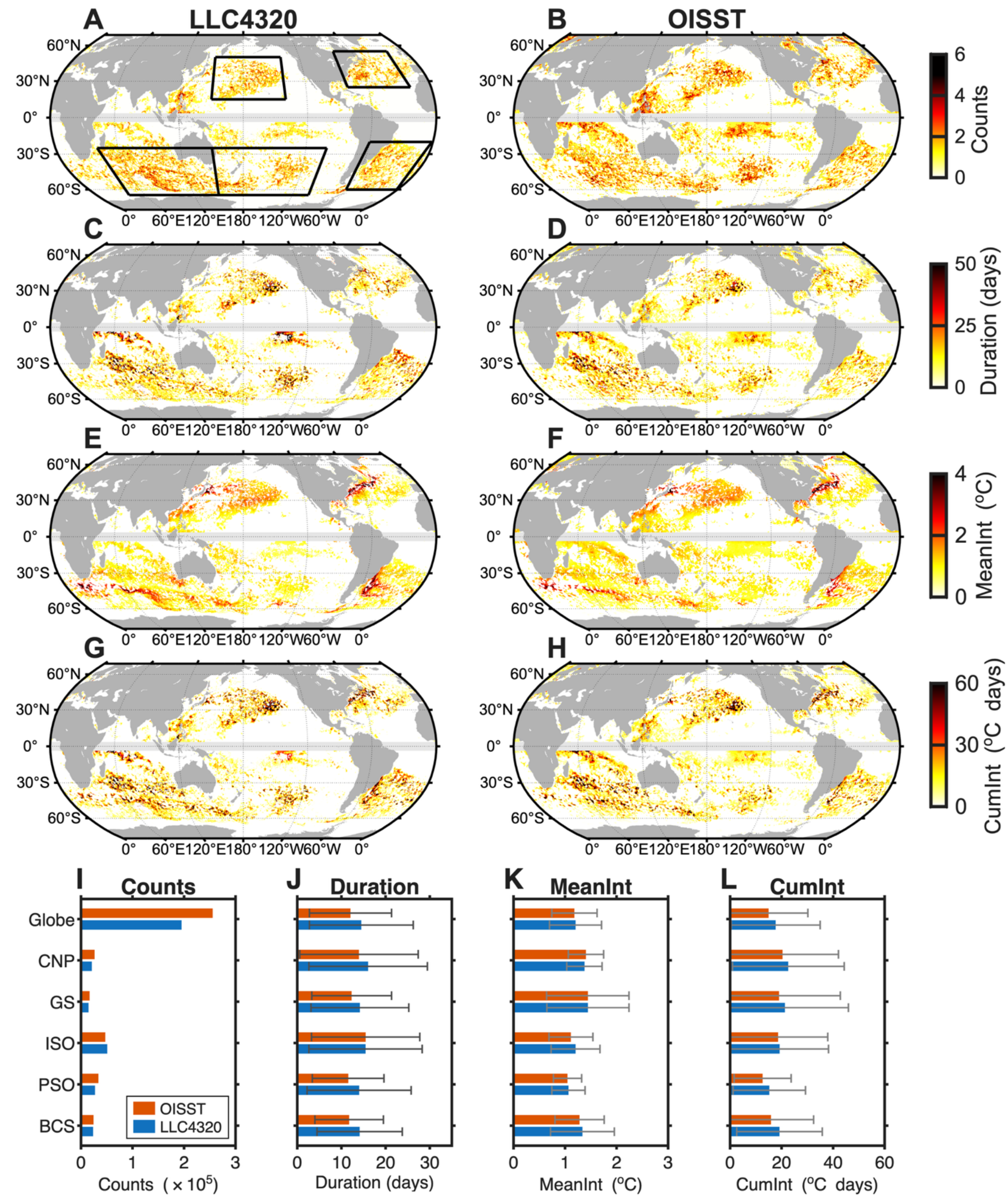

Fig. S2. A comparison of MHWs on each grid point. The spatial distributions of the (A, B) counts, (C, D) duration (days), (E, F) mean intensity (°C), and (G, H) cumulative intensity (°C days). The chart panels (I, J, K, L) show the corresponding MHW parameters over the globe and the five subregions (black boxes).

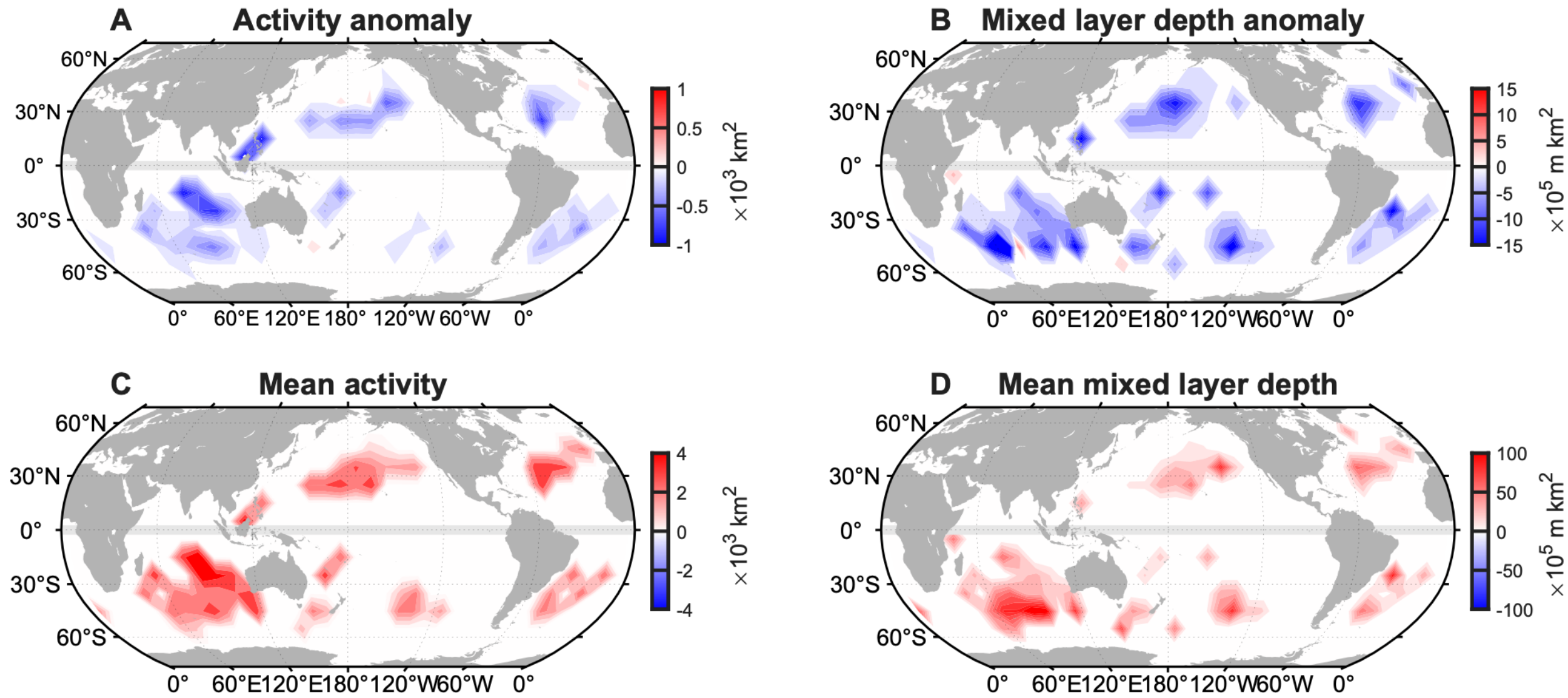

Fig. S3. Integrated submesoscale activity and mixed layer depth during Qnet-dominated MHWs. (A) Submesoscale activity anomaly; (B) Mixed layer depth anomaly; (C) Mean submesoscale activity; (D) Mean mixed layer depth. The fields are also averaged on 10º×10º grids over the globe. The mixed layer depth is defined as the depth at which the density difference reaches 0.03 kg m^{-3} relative to the value at 10 m depth.

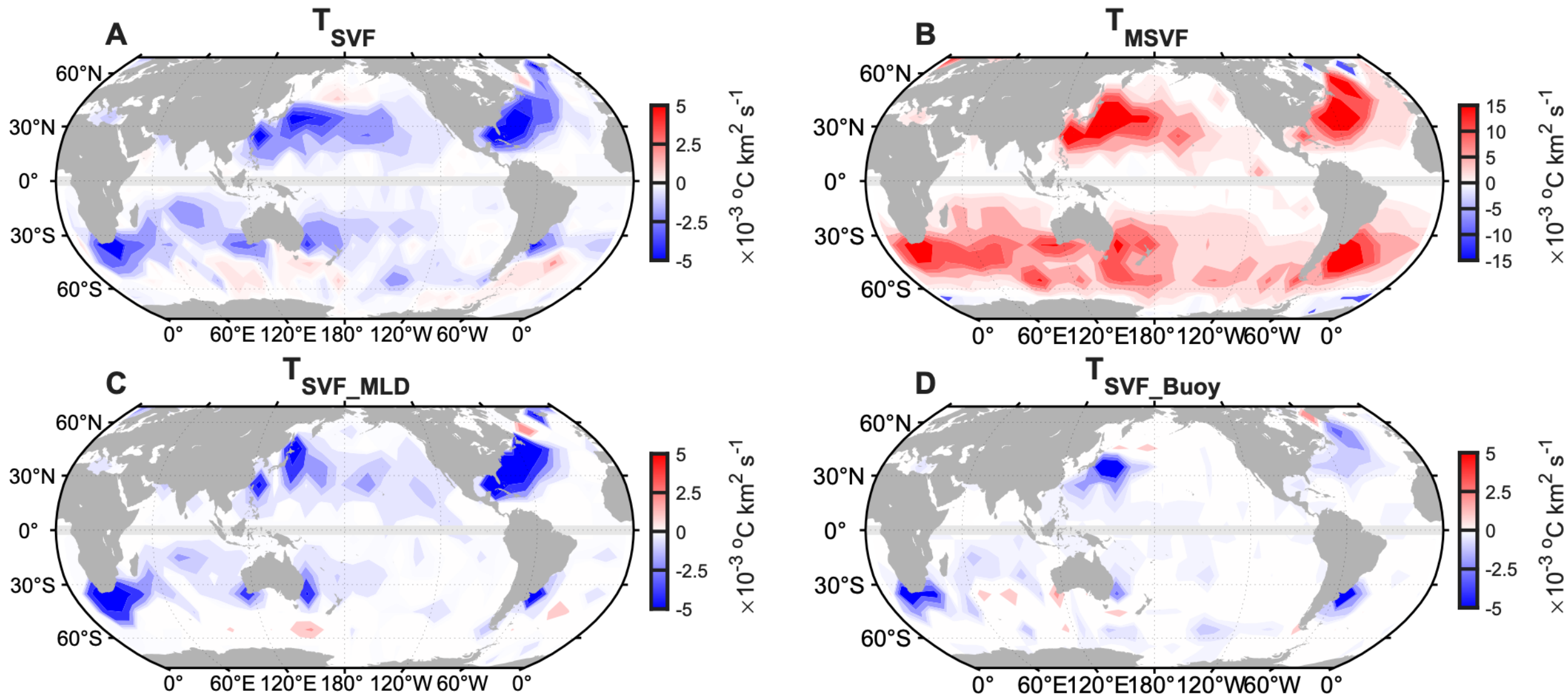

Fig. S4. Integrated submesoscale-induced vertical heat flux during MHWs from the GLORYS data combined with the Fox-Kemper et al. *(36)* submesoscale parameterization: (A) the vertical flux that excludes the mean flux, T_{SVF}; (B) the mean flux, T_{MSVF}; (C) the vertical heat flux due to the change of the mixed layer depth (i.e., the horizontal buoyancy gradient and temperature gradient are processed with 60-day moving averaging); (D) the vertical heat flux due to the change of the buoyancy and temperature gradients (i.e., the mixed layer depth is processed with 60-day moving averaging). The fluxes are averaged on $10^o \times 10^o$ grids over the globe.
